# Assessment of a novel commercial large field of view phantom for comprehensive MR imaging quality assurance of a 0.35T MRgRT system

**DOI:** 10.1002/acm2.13535

**Published:** 2022-02-23

**Authors:** Benjamin C. Lewis, Jaeik Shin, Borna Maraghechi, Benjamin Quinn, Mike Cole, Enzo Barberi, Jin Sung Kim, Olga Green, Taeho Kim

**Affiliations:** ^1^ Department of Radiation Oncology Washington University School of Medicine St. Louis Missouri; ^2^ Department of Radiation Oncology Yonsei University College of Medicine Seoul Republic of Korea; ^3^ Modus Medical Devices Inc. London Ontario Canada

## Abstract

Consistent quality assurance (QA) programs are vital to MR‐guided radiotherapy (MRgRT), for ensuring treatment is delivered accurately and the onboard MRI system is providing the expected image quality. However, daily imaging QA with a dedicated phantom is not common at many MRgRT centers, especially with large phantoms that cover a field of view (FOV), similar to the human torso. This work presents the first clinical experience with a purpose‐built phantom for large FOV daily and periodic comprehensive quality assurance (QUASAR™ MRgRT Insight Phantom (beta)) from Modus Medical Devices Inc. (Modus QA) on an MRgRT system. A monthly American College of Radiology (ACR) QA phantom was also imaged for reference. Both phantoms were imaged on a 0.35T MR‐Linac, a 1.5T Philips wide bore MRI, and a 3.0T Siemens MRI, with T1‐weighted and T2‐weighted acquisitions. The Insight phantom was imaged in axial and sagittal orientations. Image quality tests including geometric accuracy, spatial resolution accuracy, slice thickness accuracy, slice position accuracy, and image intensity uniformity were performed on each phantom, following their respective instruction manuals. The geometric distortion test showed similar distortions of –1.7 mm and –1.9 mm across a 190 mm and a 283 mm lengths for the ACR and MRgRT Insight phantoms, respectively. The MRgRT Insight phantom utilized a modulation transform function (MTF) for spatial resolution evaluation, which showed decreased performance on the lower B_0_ strength MRIs, as expected, and could provide a good daily indicator of machine performance. Both the Insight and ACR phantoms showed a match with scan parameters for slice thickness analysis. During the imaging and analysis of this novel MRgRT Insight phantom the authors found setup to be straightforward allowing for easy acquisition each day, and useful image analysis parameters for tracking MRI performance.

## 1 INTRODUCTION

Image‐guided radiotherapy (IGRT) has become the standard of care for small and mobile targets in radiation therapy, especially for high dose rate and stereotactic treatments.^1^, ^2^ Disease local control rates and normal tissue complication rates have been further improved by the integration of radiotherapy treatment units and magnetic resonance imaging (MRI) systems to produce commercial MR‐guided radiotherapy (MRgRT) systems.^3^, ^4^, ^5^ These units provide significant improvements in soft tissue imaging and real‐time tumor tracking without additional ionizing radiation dose to the patient.^6^

The delivery of high‐quality treatment and adaptation in MRgRT is dependent on accurate MRI localization, voxel sizing, and distortion correction.^7^ These requirements are complicated by the intrinsic spatial distortion and magnetic field inhomogeneity in peripheral regions of the imaging field of view (FOV).^8^, ^9^ Currently, the standard American College of Radiology (ACR) guidance for MRI accreditation has been adopted for testing MRI system performance on MR‐Linac systems and is the method recommended by AAPM Task Group 284, using the ACR Large MRI Phantom.^10^ This quality assurance (QA) methodology utilizes a phantom with a length of 148 mm and a diameter of 190 mm, with multiple reference structures incorporated throughout the volume. However, MRgRT is often used for treatment of disease in the abdominothoracic region, where large periodic motion is present and the standard imaging FOV can cover a 350 × 350 mm^2^ slice area and an imaging length of 400 mm.^11^, ^12^, ^13^ The small volume of the ACR phantom combined with its rigid setup cradle makes imaging and analysis at the periphery of the MRI field difficult to achieve. In addition to size limitations, the presence of only one test structure for each image metric would require multiple acquisitions at different locations to gather a complete dataset. The ACR phantom is also recommended only for monthly imaging QA, presenting a need for a QA system to assess imaging accuracy and uniformity on a daily basis.

Other phantoms have been clinically used for MRgRT imaging QA, such as the ViewRay daily QA phantom, the now discontinued Fluke 76–907 uniformity and linearity phantom, and custom in‐house phantoms made by various institutions. The ViewRay phantom also uses a rigid setup cradle for positioning making consistent setup across the FOV difficult. It also is primarily for dosimetric QA, with openings for ionization chambers, and only a few large alignment structures, insufficient for large FOV imaging QA. The Fluke phantom provides a grid of cylinders covering a 288 × 288 mm^2^ area in a slab type phantom that can provide geometric integrity data over a larger FOV with three setup orientations without a rigid setup cradle. This phantom only provides geometric integrity and a flood field over this FOV and has no other QA test structures. In house phantoms are difficult to provide on a large scale to all institutions that would like to use them, and do not have the full array of test structures provided by the ACR phantom. Some examples of these phantoms include a radiation and imaging isocenter alignment phantom from Dorsch et al., and a large FOV modular geometric integrity phantom from Slagowski et al.^14^, ^15^

This study presents the first clinical experience with a novel commercial daily and periodic QA phantom for radiotherapy MRI and MRgRT systems (QUASAR™ MRgRT Insight Phantom [beta], Modus Medical Devices Inc. [Modus QA], London, Ontario, Canada), imaged on a 0.35T MRgRT system, as well as 1.5T and 3.0T dedicated MRI systems. The Insight phantom is compared with the ACR phantom because of the long history of ACR phantom use in accordance with professional society, manufacturer, and institutional guidelines at our clinic.

## 2 METHODS

### 2.1 MRI quality assurance phantoms

Two phantoms were utilized in this work. The first is the standard ACR MRI phantom. The second phantom is a novel large FOV comprehensive quality assurance phantom (QUASAR™ MRgRT Insight Phantom [beta], hereafter referred to as “Insight”) produced by Modus QA. The Insight phantom can be utilized for both imaging and Linac QA, but only the imaging component is explored in this comparison study. Images were acquired on three systems, including a 0.35T ViewRay MRIdian MR‐Linac, a 1.5T Philips Ingenia MR‐simulator, and a 3.0T Siemens Vida MRI.

#### 2.1.1 ACR phantom

The standard ACR MRI phantom for quality control and system performance testing was used as a reference phantom. This phantom is currently used for monthly QA of all three MRI systems presented in this work. T1‐weighted (T1w) and T2‐weighted (T2w) images were acquired with the ACR scan parameters. On the 0.35T MR‐Linac images were acquired with the anterior and posterior array coils in place in MR‐QA mode. The ACR phantom was placed into an in‐house holder for improved setup accuracy. On the 1.5T MR‐simulator, images were acquired with the head coil and a second set of images with the anterior and posterior array coils in place. On the 3.0T MRI, images were again acquired with both the head coil and with the anterior and posterior array coils in place. ACR phantom scan parameters for all three systems are shown in Tables 1 and 2.

**TABLE 1:** T1‐weighted MRI scan parameters for the ACR and Insight phantoms on the 0.35T MR‐Linac, 1.5T MR‐simulator, and 3.0T MRI scanners

	0.35T MR‐Linac		1.5T MR‐simulator		3.0T MRI	
	ACR	Insight	ACR	Insight	ACR	Insight
TR (ms)	500	500	500	500	500	500
TE (ms)	20	20	20	20	20	20
Flip Angle (°)	90	90	90	90	90	90
FOV (mm^3^)	250 × 250 × 105	448 × 448 × 36	250 × 250 × 105	448 × 448 × 32	250 × 250 × 105	448 × 448 × 33
Matrix	256 × 256 × 11	448 × 448 × 7	256 × 256 × 11	448 × 448 × 5	256 × 256 × 11	448 × 448 × 11
Resolution (mm^3^)	0.98 × 0.98 × 5	1 × 1 × 3	0.98 × 0.98 × 5	1 × 1 × 3	0.98 × 0.98 × 5	1 × 1 × 3
Slice gap (mm)	10	6	10	8	10	3
rBW (Hz/Px)	78	78	109	109	130	130
NSA	25	25	1	1	1	1
Acquisition time (min)	53.33	93.37	4.3	7.68	2.15	3.75

**TABLE 2:** T2‐weighted MRI scan parameters for the ACR and Insight phantoms on the 0.35T MR‐Linac, 1.5T MR‐simulator, and 3.0T MRI scanners

	0.35T MR‐Linac		1.5T MR‐simulator		3.0T MRI	
	ACR	Insight	ACR	Insight	ACR	Insight
TR (ms)	2000	2000	2000	2000	3770	3830
TE (ms)	20	20	20	20	20	20
Flip Angle (°)	90	90	90	90	160	144
FOV (mm^3^)	250 × 250 × 105	448 × 448 × 36	250 × 250 × 105	448 × 448 × 32	250 × 250 × 160	448 × 448 × 51
Matrix	256 × 256 × 11	448 × 448 × 7	256 × 256 × 11	448 × 448 × 5	256 × 256 × 17	448 × 448 × 17
Resolution (mm^3^)	0.98 × 0.98 × 5	1 × 1 × 3	0.98 × 0.98 × 5	1 × 1 × 3	0.98 × 0.98 × 5	1 × 1 × 3
Slice gap (mm)	10	6	10	8	10	3
rBW (Hz/Px)	78	78.0	109	109	201	201
NSA	9	9	1	1	1	1
Acquisition time (min)	76.83	134.43	8.53	15.27	3.35	5.83

#### 2.1.2 Insight phantom

**FIGURE 1:**
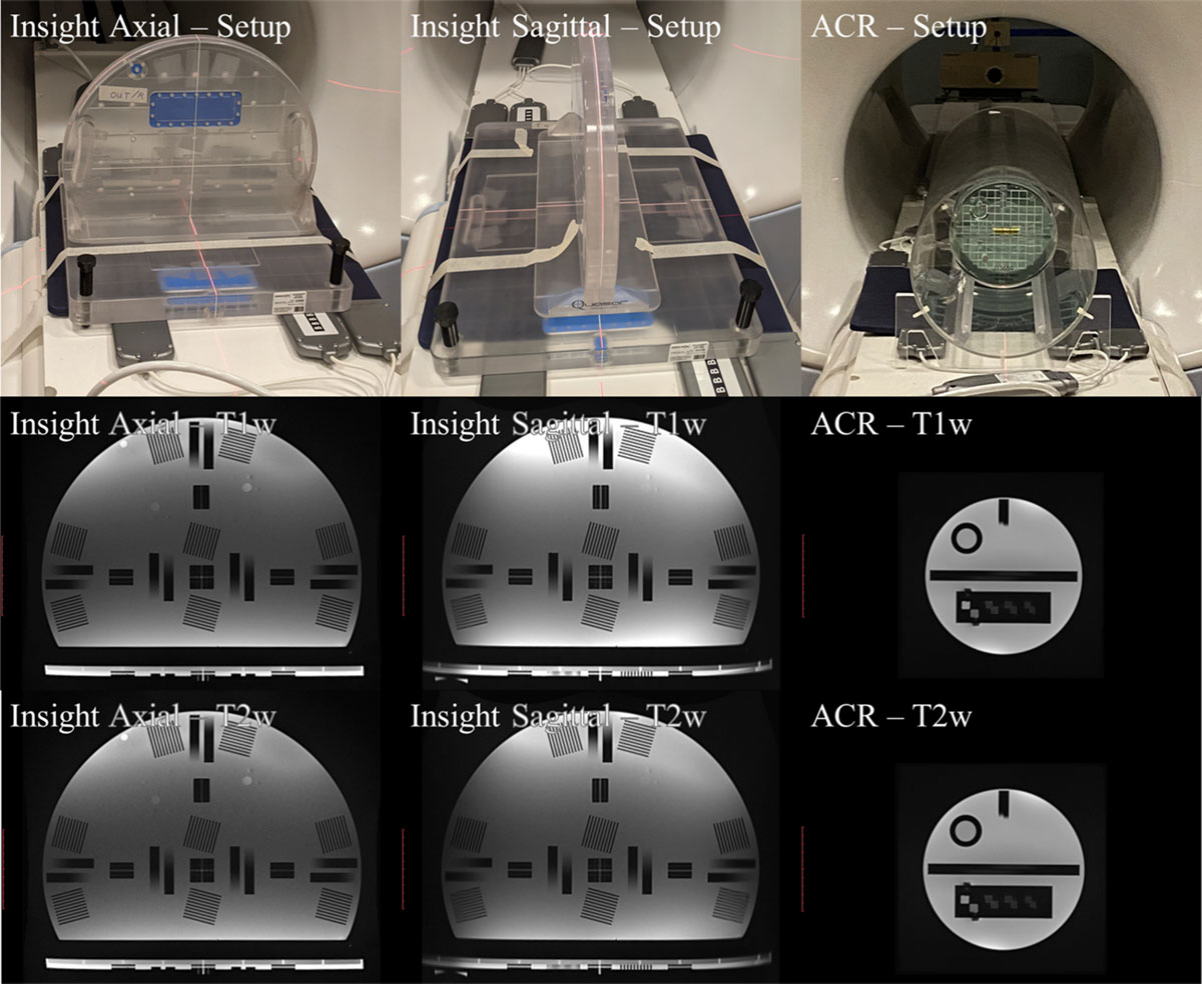
Setup, T1‐weighted, and T2‐weighted images for the Insight phantom in the axial and sagittal position, and the ACR phantom in 0.35T MRgRT system. MRI images are displayed at the same scale size

**FIGURE 2:**
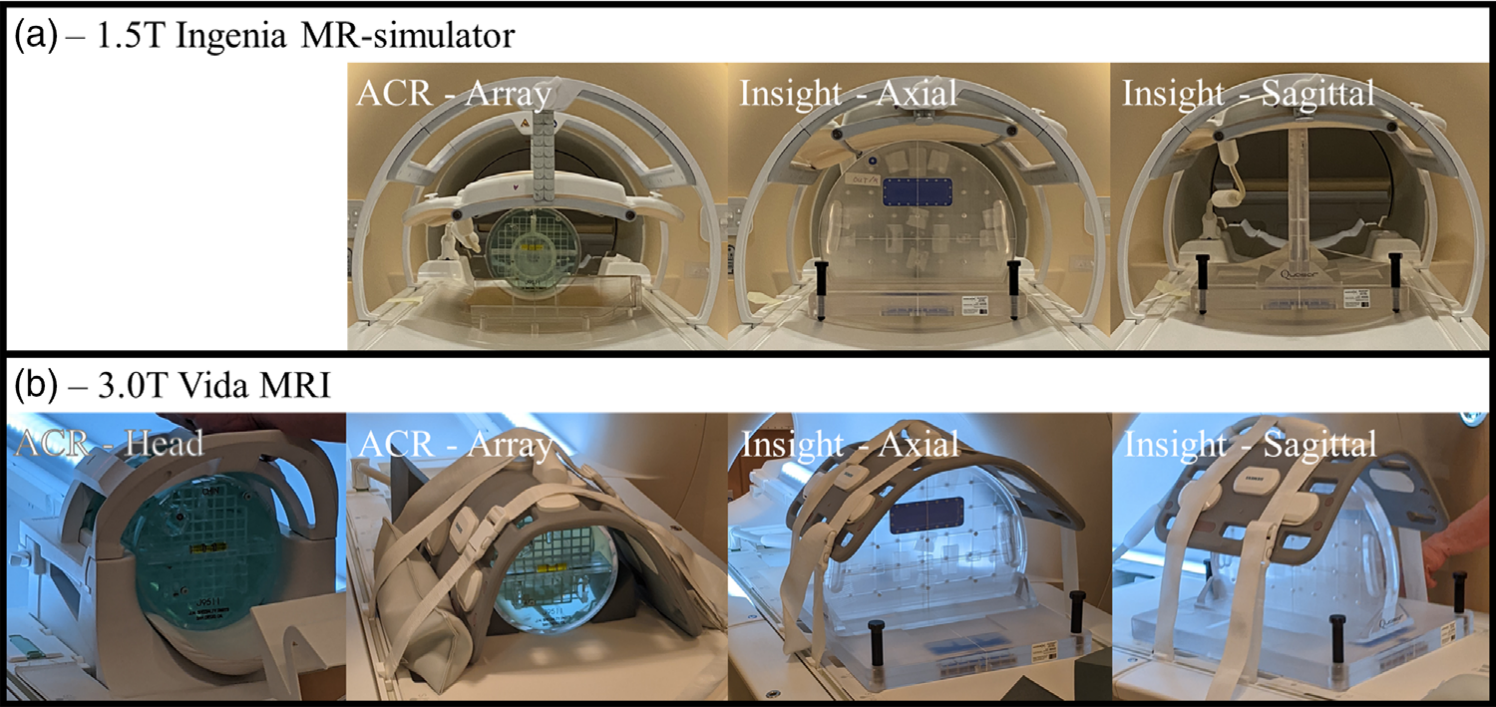
Setup images for the ACR phantom in the head coil and array coils, and the Insight phantom setup in both the axial and sagittal orientations with array coils in place on the 1.5T MR‐simulator (a) and 3.0T MRI (b) systems

The Insight phantom was imaged in both the sagittal and axial setup positions. T1w and T2w images were acquired with the ACR scan parameters but with a larger FOV to encompass the full phantom in the imaging window. On the 0.35T MR‐Linac images were acquired with the anterior and posterior array coils in place in MR‐QA mode. The 1.5T MR‐simulator and the 3.0T MRI images were acquired with the anterior and posterior array coils in place. Tables 1 and 2 show the scan parameters for T1w and T2w images, respectively. Figure 1 shows setup, T1w, and T2w images for the Insight and ACR phantoms on the 0.35T MR‐Linac. Figure 2 shows setup images for the ACR with both coils and the Insight phantom in both orientations on the 1.5T MR‐simulator and the 3.0T MRI.

### 2.2 Image analysis

#### 2.2.1 ACR phantom

ACR phantom image analysis was done manually following the ACR phantom test guidance for the MRI accreditation program.^16^ This included the following five tests:

1. Geometric accuracy measuring the grid from left to right, top to bottom, lower left corner to top right corner, and top left corner to bottom right corner. The phantom is 190 mm long in each direction.
2. High‐contrast spatial resolution using visual inspection of hole array pairs with different center‐to‐center separation and a slight staggering of rows and columns. Partial volume averaging of the holes will result in blurring of hole arrays that do not align with the display matrix.
3. Slice thickness accuracy using crossed signal ramps with known slopes. The ramps should appear the same length on the slice and have length ten times that of the slice thickness. Right or left tilt of the phantom will result in mismatched slope lengths.
4. Slice position accuracy using the crossed 45° wedges aligned to the prescribed position of image slice 1. Correct slice positioning will result in bars of equal length, while incorrect slice positioning will result in the left or right bar being longer depending on the direction of displacement.
5. Image intensity uniformity utilizing the large uniformity region. Manual adjustment of the image window and level is used to find 1 cm^2^ ROIs with the greatest and lowest mean signal intensities. Percent integral uniformity (PIU) is calculated using the following equation: $PIU\;=\;100\times (1-\{\frac{SI_{high}-SI_{low}}{SI_{high}+SI_{low}}\})$

**FIGURE 3:**
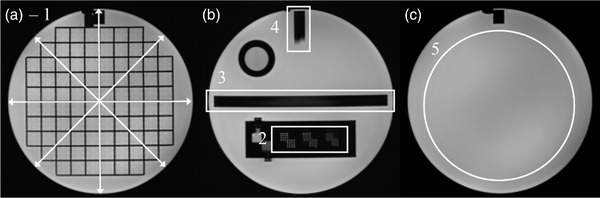
Image analysis regions for the ACR phantom. Image (a) shows the geometric accuracy grid with arrows indicating the four measured distances (1). Image (b) includes the high‐contrast spatial resolution test (2), slice thickness test (3), and slice position accuracy test (4). Image (c) includes the image intensity uniformity region (5)

The analysis was performed by a single operator for all scans. Figure 3 displays the test regions for the ACR phantom.

#### 2.2.2 Insight phantom

A similar set of tests was performed using the inserts available on the Insight phantom, following the instructions provided by Modus QA, based on recommendations from IEC 62464‐1.^17^ These five tests included the following:

1. Geometric accuracy measuring the grid from left to right, top to bottom, lower left corner to top right corner, and top left corner to bottom right corner. The measurement distance is 200 mm long for the horizontal and vertical directions, and 282.9 mm long for the angled measurements. Peripheral measurements were also performed in the left, right, and top peripheral sections, which have a length of 100 mm.
2. High‐contrast spatial resolution using a circular ROI in the four pairs of resolution test objects, for the frequency and phase encoding directions, and calculating a representative value from the modulation transfer function (MTF) using 7.5 cm^2^ circular ROIs.^18^
3. Slice thickness accuracy using the five slice thickness ramp pairs. Line profiles are generated along each ramp pair and the inflection points between the ramp's saturated top, sloped transition, and saturated bottom are used to calculate the thickness.
4. Slice position accuracy using the plane alignment structures, providing a qualitative but not quantitative indicator of alignment. The value is presented as a percent of marker lines visible over the four acquisitions on each system.
5. Image intensity uniformity using the average of standard deviations for four 15 cm^2^ circular ROIs placed in uniform signal‐producing regions. This was performed with both the array receiver coil and the body receiver coil.

**FIGURE 4:**
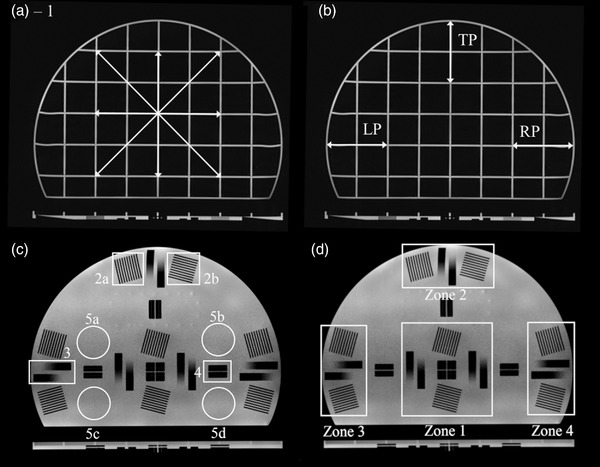
Image analysis regions for the Insight phantom. Image (a) indicates the geometric accuracy grid with four arrows indicating the measured distances. Peripheral geometric accuracy regions (b) include the left periphery (LP), right periphery (RP), and top periphery (TP). Image (c) includes the high‐contrast spatial resolution test (2a and b), slice thickness test (3), slice position accuracy test (4), and image intensity uniformity (5a–d). The Insight phantom includes four test regions (d): the center, center top, left side, and right side

Tests 2–5 were performed in four separate regions of the phantom, located at the phantom center (zone 1), anterior edge (zone 2), left‐side edge (zone 3), and right‐side edge (zone 4). Test regions are shown in Figure 4. All tests are summarized in Table 3.

**TABLE 3:** Summary of image analysis tests for the ACR and Insight phantoms

Test name	ACR phantom method	Insight phantom method
Geometric accuracy	Measure phantom diameter in four directions on the grid portion of the phantom (diameter = 190 mm)	Measure grid spacing in four directions including 200 mm horizontal, 200 mm vertical, 283 mm diagonals, and three peripheral regions 100 mm in length
High‐contrast spatial resolution	Frequency and phase encoding direction resolution hole array pairs	–
Periodic spatial resolution	–	Modulation transfer function calculation within 7.5 cm^2^ circular ROIs drawn over frequency and phase encoding specific line pairs
Slice thickness accuracy	One pair of signal ramps	Five pairs of signal ramps
Slice position accuracy	Using separation of the two edges of a 45° wedge pair	–
Setup position accuracy	–	Four sets of plane alignment structures with 1.5 mm and 3.0 mm channels
Image intensity uniformity	Percent integral uniformity from high‐ and low‐intensity regions of a large uniformity region	Uniformity expressed as the average of standard deviations from four 15 cm^2^ circular ROIs

## 3 RESULTS

### 3.1 Geometric accuracy

The ACR phantom had a maximum geometric error of –1.7 mm across the horizontal direction, occurring for the T2w acquisition on the 0.35T MR‐Linac with the array coil in place. The maximum geometric error with the head coil in place was –1.5 mm, occurring in the horizontal direction on the T2w scan acquired on the 1.5T MR‐simulator. The maximum geometric error for the Insight phantom was –2.3 mm in the top left to bottom right diagonal in the axial orientation on the T1w image acquired on the 1.5T MR‐simulator. Sagittal scans of the Insight phantom had a maximum geometric error of –1.1 mm, also in the top left to bottom right diagonal of the T2w scan on the 1.5T MR‐simulator. Table 4 includes all geometric error values across all scans and directions. Visual inspection shows deformation of the Insight phantom in the peripheral grid segments. The error values for the peripheral grid segment lengths are shown in Table 5.

**TABLE 4:** Geometric accuracy error from the expected measurement length for the ACR and Insight phantoms on all three systems, with array and head coils in place, and T1‐weighted (T1w) and T2‐weighted (T2w) scans

System	Phantom	Orientation	Scan	Horizontal error (mm)	Vertical error (mm)	Diagonal BL to TR error (mm)	Diagonal BR to TL error (mm)
0.35T MR‐Linac	ACR	Axial—array	T1w	–0.7	–0.3	–0.8	–0.6
			T2w	–1.7	0.0	–0.8	–1.1
	Insight	Axial—array	T1w	–0.8	–0.2	0.6	–0.3
			T2w	–0.2	–0.2	–0.3	––0.3
		Sagittal—array	T1w	–0.2	–0.2	0.2	–1.1
			T2w	–0.5	–0.5	–0.2	–0.7
1.5T MR‐simulator	ACR	Axial—head	T1w	–0.7	–1.0	–0.3	–0.6
			T2w	–1.5	–0.4	–1.0	–0.8
		Axial—array	T1w	–1.3	–0.3	–1.6	–1.2
			T2w	–0.7	–0.3	–0.8	–0.6
	Insight	Axial—array	T1w	–0.4	–0.4	–0.6	–2.3
			T2w	0.1	–0.5	–0.2	–2.9
		Sagittal—array	T1w	0.1	–0.5	–0.7	–0.2
			T2w	0.8	–0.5	–1.11	–1.11
3.0T MRI	ACR	Axial—head	T1w	–0.3	0.0	–0.1	–0.8
			T2w	–0.7	0.7	–0.3	0.2
		Axial—array	T1w	–1.4	–0.4	–0.4	0
			T2w	–0.3	0	–0.3	–0.3
	Insight	Axial—array	T1w	–0.8	–0.2	0.6	–0.3
			T2w	–0.2	–0.2	–0.3	–0.3
		Sagittal—array	T1w	–0.2	–0.2	0.2	–1.1
			T2w	–0.5	–0.5	–0.2	–0.7

BL, bottom left; BR, bottom right; TL, top left; TR, top right.

**TABLE 5:** Geometric accuracy error from the expected measurement length for the peripheral regions of the Insight phantom on T1‐weighted (T1w) and T2‐weighted (T2w) scansl

System	Orientation	Scan	LP	RP	TP
0.35T MR‐Linac	Axial	T1w	–2.1	–2.1	–2.7
		T2w	–2.1	–2.1	–3.3
	Sagittal	T1w	–4.6	–1.7	–2.1
		T2w	–2.1	0.6	–2.7
1.5T MR‐simulator	Axial	T1w	–2.1	–0.2	–1.5
		T2w	–2.1	–0.8	–4.5
	Sagittal	T1w	–5.2	1.0	–1.5
		T2w	–5.2	1	–1.5
3.0T MRI	Axial	T1w	–2.9	–2.3	–0.4
		T2w	–1.7	–2.3	–0.4
	Sagittal	T1w	2.7	–4.8	–2.3
		T2w	0.8	–4.2	–1.7

LP, left peripheral; RP, right peripheral; TP, top periphera.

### 3.2 High‐contrast and periodic spatial resolution

**FIGURE 5:**
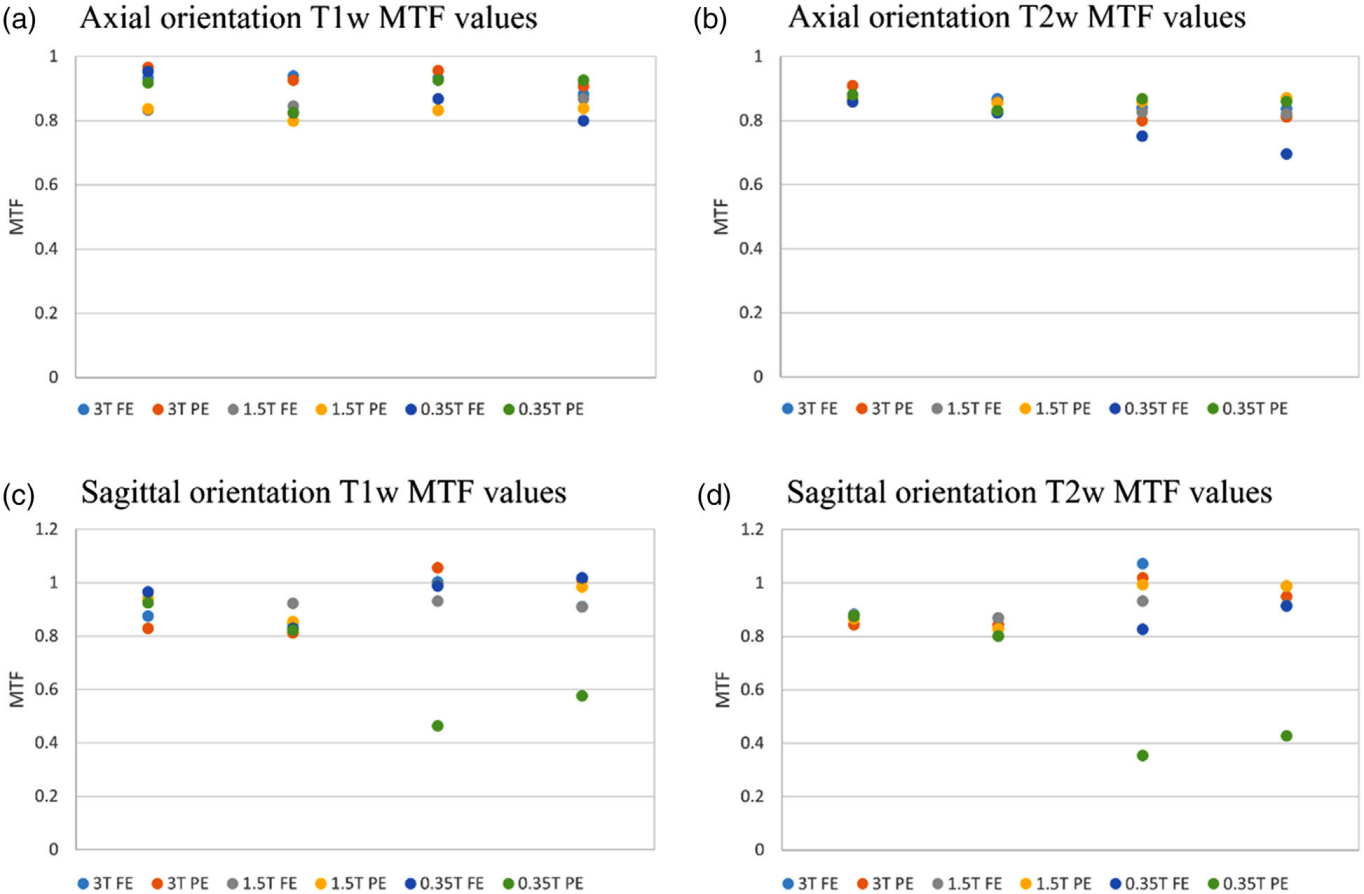
MTF values for the Insight phantom in the axial and sagittal orientations, T1‐weighted and T2‐weighted acquisitions, for the frequency encoding (FE) and phase encoding (PE) directions. Images were acquired on a 3.0T Siemens Vida MRI, a 1.5T Philips Ingenia MRI, and a 0.35T ViewRay MRIdian MR‐Linac. Zones 3 and 4 in some sagittal images were affected by both in‐plane and through‐slice distortion and as a result may not reflect valid MTF values

The high‐contrast spatial resolution region indicated a resolution of 1 mm in the frequency and phase encoding direction for all ACR phantom scans. The Insight phantom utilized MTF values for the frequency and phase encoding directions. The minimum MTF value was 0.35 for the sagittal orientation T2w acquisition on the 0.35T MR‐Linac, in zone 3. This structure and the corresponding structure in zone 4 were affected by a blurring artifact in the sagittal MRIdian images. Modus QA has listed a passing threshold of 0.8 for this phantom, as recommended in IEC 62464‐1. All MTF values are shown in Figure 5 for the frequency and phase encoding directions.

### 3.3 Slice thickness and setup accuracy

The ACR phantom showed a maximum slice thickness error of 0.9 mm, –0.2 mm, and 0.9 mm for the 3.0T, 1.5T, and 0.35T systems, respectively, with an expected slice thickness of 5.0 mm. These errors all occurred with the array coil in place. The Insight phantom showed good agreement with the expected slice thickness of 3.0 mm in most cases, with errors in zone 1 for the axial orientation being 0.10, 0.16, and 0.17 mm for the 3.0T, 1.5T, and 0.35T systems, respectively, and zone 1 sagittal orientation errors of 0.48, 0.20, and 0.13 mm. Table 6 shows the slice thickness tests for both the ACR and Insight phantoms, the Insight phantom results are presented as the average of slice thickness results in each zone.

**TABLE 6:** Slice thickness error from the expected thickness for the ACR and Insight phantoms on all three systems, with array and head coils in place, and T1‐weighted (T1w) and T2‐weighted (T2w) scans

System	Phantom	Orientation	Scan	Zone 1	Zone 2	Zone 3	Zone 4
0.35T MR‐Linac	ACR	Standard—Array	T1w	0.7	N/A	N/A	N/A
			T2w	0.9	N/A	N/A	N/A
	Insight	Axial—Array	T1w	0.17	0.1	–0.07	–0.1
			T2w	0.08	0.1	–0.02	–0.23
		Sagittal—Array	T1w	0.13	0.02	0.18	–0.06
			T2w	0.00	0.01	0.10	0.05
1.5T MR‐simulator	ACR	Standard—Array	T1w	–0.1	N/A	N/A	N/A
			T2w	0.0	N/A	N/A	N/A
		Standard—Head	T1w	–0.2	N/A	N/A	N/A
			T2w	–0.2	N/A	N/A	N/A
	Insight	Axial—Array	T1w	–0.16	–0.14	–0.05	–0.26
			T2w	–0.12	–0.12	–0.22	–0.40
		Sagittal—Array	T1w	–0.20	–0.04	–0.07	–0.05
			T2w	–0.12	–0.13	–0.04	–0.22
3.0T MRI	ACR	Standard—Array	T1w	–0.3	N/A	N/A	N/A
			T2w	0.7	N/A	N/A	N/A
		Standard—Head	T1w	–0.4	N/A	N/A	N/A
			T2w	0.9	N/A	N/A	N/A
	Insight	Axial—Array	T1w	0.10	0.17	0.17	0.22
			T2w	0.48	0.76	0.33	0.49
		Sagittal—Array	T1w	0.16	–0.19	0.44	–0.04
			T2w	0.62	0.14	0.22	0.10

*Note*: For the Insight phantom, the average slice thickness error is reported for all ramps in the measurement zone. Zones 2–4 are listed as N/A for the ACR phantom (light gray)

**FIGURE 6:**
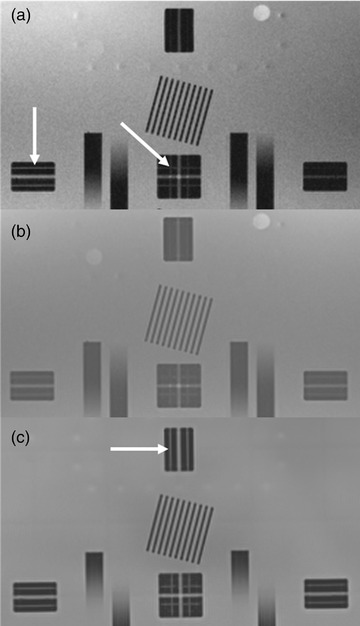
Position accuracy markers on the (a) 0.35T MR‐Linac, (b) 1.5T MR simulator, and (c) 3.0T MRI. The white arrows indicate the fine laser alignment marker that is 1.5 mm wide

The maximum slice position error was 1.4, 1.2, and 1.8 mm for the ACR phantom measured on the 3.0T, 1.5T, and 0.35T systems, respect tint="gray"ively. An initial set of scans of the Insight phantom using the system lasers for alignment found percentages of slice alignment markers visible of 92.5%, 100%, and 100% for the 3.0T, 1.5T, and 0.35T systems, respectively. Figure 6 shows example T1w images of the laser alignment marker on the (a) 0.35T MR‐Linac, (b) 1.5T MR‐simulator, and (c) 3.0T MRI.

### 3.4 Image intensity uniformity

The ACR phantom analysis had percent integral uniformity (PIU) values greater than 86.6 for all scans, with the top PIU value occurring for the T1w acquisition on the 1.5T MR‐simulator with the head coil in place. The lack of a large uniform region prevents an accurate PIU value calculation on the Insight phantom, and an average of standard deviations for four uniform ROIs of 15 cm^2^ was used in its place. The average standard deviation as a percent of average signal intensity ranged from 3.27% to 6.06%, 4.77% to 5.64%, and 12.15% to 13.54% for the 3.0T, 1.5T, and 0.35T systems, respectively, with the array coil in place. A similar analysis was performed on the ACR phantom within the uniformity region; however, the four ROIs were closer to the imaging isocenter due to the small slice area. The ACR phantom had an average of standard deviations for the for ROIs less than 1.35% of average signal intensity for all three scanners. When the body coil was used instead of the array coil on the 1.5T and 0.35T systems, the values reduced to 4.93%–6.69% and 4.06%–6.98%.

## 4 DISCUSSION

This study presents the first clinical experience with the Modus QA QUASAR™ MRgRT Insight Phantom (beta), applied to two dedicated MRI systems and a 0.35T MR‐Linac system. Phantom setup was simple and showed good alignment with the slice position identifiers, allowing for quick setup for use in a daily QA clinical situation. Manual analysis was used for this work but generating a semiautomated analysis software is feasible and would streamline daily use. Additionally, the phantom provided similar QA metrics as those provided by the standard ACR phantom, which is currently used for monthly imaging QA at our institution. The QA metrics could be measured over a much larger FOV with the Insight phantom than is available for the ACR phantom and allows for both axial and sagittal acquisition planes. This is especially important for MR‐Linac systems where geometric accuracy is needed at the image periphery for accurate localization and dose calculation during online adaptive radiotherapy. The increased FOV appeared to show a change in the geometric accuracy error values between the ACR and Insight phantoms when measuring diagonal path lengths, which were 190 mm for the ACR phantom and 282.9 mm for the Insight phantom. The diagonal errors had differences of up to 2.3 mm for the images acquired on the 1.5T MR simulator, while the horizontal and vertical errors were less than 1.5 mm across the three MRI systems. Uniformity values using the four ROI standard deviation values measured on the ACR phantom were also less than those from the Insight phantom where ROIs were further apart. The spatial resolution tests used by the phantoms are also different, with the ACR phantom utilizing high‐contrast resolution inserts allowing for determining image resolution at three different sizes, and the Insight phantom utilizing periodic spatial resolution objects derived from IEC 62464‐1 standard.^17^ In the instance of the plate stack used by the Insight phantom, the standard deviation divided by the mean signal intensity within the feature ROI has been assigned a threshold value of 0.56, corresponding to an MTF of 0.8. The two test objects aligned at a 90° angle to one another allows for assessment in the frequency and phase encoding directions and can detect changes in the resolution behavior of the system, such as anomalous values and trends over time.^18^, ^19^

The four QA feature zones of the Insight phantom allows for capturing distortion and image quality changes across the entire planar field of view, up to 40 cm, in one image acquisition, unlike other spatial integrity phantoms, which require multiple setup positions to capture those changes. This helps to reduce setup error induced variation and reduce the total required time. For the comparison of these two QA phantoms, the pulse sequence parameters followed the direction of the ACR and Modus QA guidelines according to phantom features. This resulted in different slice thickness and slice gap matched to the feature size of each phantom, which may impact the error of the associated QA parameters; however, in the daily QA setting, detection of drift in imaging parameters would not be impacted by these differences. Additionally, the Insight phantom has the potential to integrate radiation delivery QA with imaging QA, which is not possible with the ACR phantom, but was not a part of this study.

The Insight phantom has some different features compared to the ACR phantom. Due to the large area and relatively thin target structure area, a slight tilt during setup could result in partial volume averaging of the grid and structure regions. This averaging prevents image analysis in the regions that the two structures are visible. For this reason, a localizer scan is recommended for verifying alignment rather than relying on the system lasers. Additionally, the slice thickness measurement can be affected by the use of a phased‐array coil. The array coil creates a signal intensity gradient across the large field of view of the phantom, making it difficult to identify the start and end positions of the slice thickness ramps. This limitation could be alleviated through the use of automated image analysis; however, the requirement for careful alignment of the phantom and imaging space would remain. A final limitation of the Insight phantom is the acquisition time increase due to the larger field size, with total acquisition time nearly doubling. Following the recommended pulse sequence parameters for both phantoms also resulted in the exclusion of true fast imaging with steady‐state free precession (TRUFI) pulse sequence acquisitions. A TRUFI sequence can be used for target tracking and gating during MRgRT, such as on the ViewRay system, and tracking the daily performance of such sequences would be valuable for maintaining consistent high‐speed image acquisitions. Utilizing fewer signal averages would additionally reduce the total acquisition time to less than 10 min for the Insight phantom. Future works will look toward incorporating fast imaging sequence evaluation with the Insight phantom DQA program.

This work assessed the clinical utility of the Modus QA QUASAR™ MRgRT Insight Phantom (beta), using the ACR accreditation phantom as a reference for image analysis values, across three MRI units including 3.0T and 1.5T dedicated MRI systems, and a 0.35T MR‐Linac system. The Insight phantom provides a large FOV phantom with multiple acquisition orientations that are easy to accurately setup in a lightweight package. The MRgRT Insight phantom offers the ability to monitor key imaging QA parameters over a field of view relevant to radiation therapy on a daily basis, including laser and imaging isocenter alignment, B_0_ uniformity, SNR, geometric distortion, image ghosting, spatial resolution, and slice thickness. While the ACR phantom will remain a requirement for periodic certification at many sites, the time‐consuming setup process and lengthy scanning requirements make it unsuitable for daily QA. The limited extent of the ACR phantom also leaves much of the RT field of view unexamined.  In addition, the Insight phantom offers end‐to‐end verification of MR and RT isocenter coincidence, replacing the need for additional targeting phantoms.  The phantom will be provided with automated Image Quality analysis including trending and reporting that will add significant efficiency improvements to a robust QA program with daily imaging QA utilizing the insight phantom and monthly quality control with the ACR phantom.

## 5 CONCLUSION

The authors presented the first clinical experience with a novel daily and periodic quality assurance phantom for MR imaging. The large field of view allowed for assessing image quality factors at a wider range of positions relative to the current ACR phantom used in our clinic and could be aligned quickly and reliably to in‐room laser systems. The Insight phantom allowed for assessment of similar metrics to the ACR phantom, which would allow for tracking imaging changes over time at a much higher frequency than with the current practice.

## CONFLICT OF INTEREST

The authors declare no conflicts of interest.

## AUTHOR CONTRIBUTIONS

Benjamin Lewis contributed to data collection, analysis, and writing of the manuscript. Jaeik Shin contributed to data collection. Enzo Barberi, Benjamin Quinn, and Mike Cole contributed to phantom set‐up recommendations, design of data analysis, and provided technical assistance. Jin Sung Kim and Olga Green contributed to study design and supervision. Taeho Kim contributed to data collection, analysis, and supervision of the project. All authors discussed the results and contributed to the final manuscript.

## Data Availability

The data that support the findings of this study are available from the corresponding author upon reasonable request.
